# Rapid Simultaneous Determination of 43 Pesticide Residues in *Schizonepeta tenuifolia* by Gas Chromatography Mass Spectrometry

**DOI:** 10.1155/2021/8934998

**Published:** 2021-12-13

**Authors:** Guo-wei Zhou, Yong-mei Li, Chun-ni Liu, Hong-min Ren, Hai-ying Li

**Affiliations:** ^1^Chengyang District People's Hospital of Qingdao, Qingdao, Shandong 266109, China; ^2^Baoding Customs Office, Baoding 071000, China; ^3^College of Pharmacy, Hebei University, Key Laboratory of Pharmaceutical Quality Control of Hebei Province, Baoding 071002, China

## Abstract

A simple, fast, and reliable method was established for simultaneous determination of 43 pesticides in *Schizonepeta tenuifolia*. The samples were prepared using solid-phase extraction (SPE) method. Pesticides were extracted from *Schizonepeta tenuifolia* using acetonitrile, cleaned with Pesticarb/NH_2_, and eluted by mixed solvents of acetonitrile and toluene (3 : 1, v/v). Selected pesticides were identified using DB-35MS capillary column and detected by gas chromatography mass spectrometry. Samples were quantified by external standard method. Recoveries for the majority of pesticides at spike levels of 0.2, 0.5, and 1 mg kg^−1^ ranged between 70 and 120% (except for Chlorothalonil, Thiamethoxam, and Dicofol), and the relative standard deviations (RSDs *n* = 6) were 1.32%–13.91%. Limits of detection (LODs) were 0.0011–0.0135 mg kg^−1^, whereas limits of quantification (LOQs) were 0.0038–0.0451 mg kg^−1^. The satisfactory accuracy and precision, in combination with a good separation and few interferences, have demonstrated the strong potential of this technique for its application in *Schizonepeta tenuifolia* analysis.

## 1. Introduction

Chinese herbal medicines (CHMs) have been widely used as a means of medication for their mild pharmaceutical effects and minimum side effects [1]. What is more, they have been considered to be gentle, nontoxic, and even harmless mainly because of their natural origin [2]. On account of the increasing requirements, a large amount of pesticides are applied for reducing loss from insects and diseases, which could lead to a high risk of contamination from agricultural chemicals [3]. What is worse, the bioaccumulation and persistent character of pesticides also pose a threat to human health as well as the environment [4–6]. Therefore, pesticides residue determination in CHMs plays an important role in market monitoring, environmental research, risk assessment of dietary intakes, and especially in the exportation of CHMs worldwide.

*Schizonepeta tenuifolia* is one of the most frequently used crude drugs for oriental medicine in China, Korea, Japan, and other Asian areas. It plays an important role in agricultural production and people's living. Pharmacological studies and clinical practice have proven that it is used as an antipyretic, analgesic, antipathogenic microorganisms, anti-inflammatory, antioxidation, and hemostasis drug for the treatment of colds, headaches, measles, rubella, sores, etc. [7]. Like other crops, *Schizonepeta tenuifolia* is susceptible to insect and disease attacks both in field and storage, so pesticides are widely used for their protection. Hence, it is significative to monitor pesticide residues in *Schizonepeta tenuifolia.*

Pesticides can be mainly categorized into five classes, namely, organochlorine pesticides (OCPs), organophosphorus pesticides (OPPs), pyrethroid pesticides (PYRs), carbamate pesticides (CBs), and other type of pesticides. Only a few analytical methods for the determination of pesticide residues in *Schizonepeta tenuifolia* have been described in recent literature [3, 8, 9]. Yi and Lu [10] reported a multiresidue method for the determination of 17 pesticides with only OCPs and OPPs in *Schizonepeta tenuifolia.* Yet these methods are showing their limits about pesticide varieties. In the case of treatment with sulfuric acid, it cannot be applied to the analysis of the pesticides that decompose easily under strong acidic conditions, such as PYRs.

Mass spectrometry (MS) is a good multiresidue analysis technique for its universal property and higher sensitivity. It not only can effectively discriminate the signals between analyte and impurities, but also can be identified and quantify the results via the selected ion monitoring (SIM) spectra. Therefore, it can be used to detect different types of pesticides in a variety of different substances, such as fruits [11], vegetables [12], spices [13], grape wine [14], or Chinese teas [15]. To eliminate the interferences of matrixes and keep the chromatographic system in good working order, an effective sample preparation process for different detected objects is necessary. There are many pretreatment techniques for the extraction and clean-up of pesticides in CHMs. Fu et al. [16] reported a multiresidue method using gas chromatography mass spectrometry for the determination of 201 pesticides in different CHMs. Yet, these methods are not capable of removing the complex coextractives that may interfere with the detection of the analytes of *Schizonepeta tenuifolia*. Besides, some of these technologies are relatively time-consuming and require large volumes of organic solvents. This is hazardous to human health and causes serious pollution problems. Therefore, the achievement of a good separation and few interferences from *Schizonepeta tenuifolia* samples is considered to be a difficult, in some respect even more challenging task.

To the best of our knowledge, this is the first report on the multiresidue analysis by gas chromatography mass spectrometry in *Schizonepeta tenuifolia*. A multiresidue extraction method using the orthogonal test coupled with dispersive SPE technique was firstly proposed to determine 43 pesticide residues in *Schizonepeta tenuifolia*, including seventeen OCPs, nine OPPs, five PYRs, two CBs, and ten other pesticides. An additional purpose was to find the best performance of the different pretreatments. In this study, the extraction method was optimized using an orthogonal test of L_9_(3^4^), according to different extraction solvent, extraction solvent volume, and the extraction time. Meanwhile, the type of sorbents and eluents which affected the efficiency of SPE was investigated. A simple, fast, and reliable method was finally established for simultaneous determination of 43 pesticides in *Schizonepeta tenuifolia*. Moreover, this might be helpful for detecting pesticide residues in other CHMs in addition.

## 2. Materials and Methods

### 2.1. Chemicals and Materials

Pesticide standards including dichlorvos, carbofuran, methamidophos, ethoprophos, omethoate, phorate, hexachlorobenzene, *α*-BHC, monocrotophos, quintozene, *γ*-BHC, dimethoate, acetochlor, *β*-BHC, prometryn, *δ*-BHC, metalaxyl, chlorothalonil, aldrin, chlorpyrifos, parathion, dicofol, pendimethalin, *cis*-heptachlor epoxide, *trans*-heptachlor epoxide, *cis*-chlordane, *trans*-chlordane, thiamethoxam, *pp'*-DDE, dieldrin, fludioxonil, *op'*-DDT, nitrofen, *pp'*-DDD, *β*-endosulfan, propiconazole, *pp'*-DDT, bifenthrin, fenpropathrin, cyhalothrin, cypermethrin, fenvalerate, and difenoconazole were purchased from Agro-Environment Protection Institute (Tianjin, China). The individual stock standard solution of each pesticide is 100 mg L^−1^. This solution was further diluted with n-hexane to give working solution concentrations of 50, 100, 200, 500, and 1000 *μ*g L^−1^, respectively, and all solutions were stored at 4°C. Acetonitrile, petroleum ether, methylbenzene, and acetone (HPLC grade) were supplied by Tianjin Chemical Reagents Co. (Tianjin, China). Florisil (250 mg mL^−1^), TPT (2000 mg/12 mL), and Pesticarb/NH_2_ (500 mg/500 mg/6 mL) were purchased from Tianjin Agela Technologies Co. (Tianjin, China).

### 2.2. GC-MS Conditions

The chromatographic system was a QP-2010Plus gas chromatograph coupled with a mass spectrometer and a AOC-20i autosampler (Shimadzu instrument Co., Japan). All 43 pesticides were separated on DB-35 ms capillary column (30 m × 0.25 mm, 0.25 *μ*m film thickness; Agilent Co., USA). The oven temperature was programmed as follows: the initial column temperature is 50°C, increased at 45°C min^−1^ to 130°C, then at 10°C min^−1^ to 190°C, then at 4°C min^−1^ to 240°C, hold for 1 min, finally at 15°C min^−1^ to 305°C, hold for 10 min. The inlet temperature was set at 290°C. The injection volume was 1 *μ*L with a splitless mode. Helium gas (99.999% purity) was chosen as the carrier gas with a constant flow of 1.1 mL min^−1^. The mass spectrometer was operated at 70 eV in electron impact (EI) mode with an ion source temperature of 230°C. The transmission line temperature and solvent delay time were 280°C and 5 min, respectively. Under the same chromatographic conditions, target and qualifier abundances were determined by injection of individual pesticide standards in full-scan mode with the mass/charge ratio ranging from *m*/*z* 50 to 500. Analysis was performed in the selected ion monitoring mode (SIM) and the mass fragment with the highest intensity was used for quantification, while two or three are selected as the qualifier ions from the remaining fragments.

**Figure 1:**
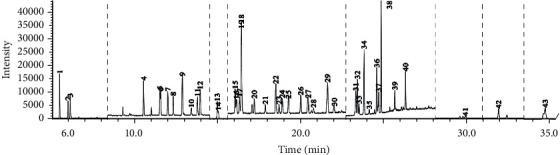
GC-MS-SIM chromatogram of standard mixture of the 43 pesticides (0.5 mg L^−1^): 1: dichlorvos, 2: carbofuran, 3: methamidophos, 4: ethoprophos, 5: omethoate, 6: phorate, 7: hexachlorobenzene, 8: *α-*BHC, 9: monocrotophos, 10: quintozene, 11: *γ*-BHC, 12: dimethoate, 13: acetochlor, 14: *β*-BHC, 15: prometryn, 16: *δ*-BHC, 17: metalaxyl, 18: chlorothalonil, 19: aldrin, 20: chlorpyrifos, 21: parathion, 22: dicofol, 23: pendimethalin, 24: *cis*-heptachlor epoxide, 25: *trans*-heptachlor epoxide, 26: *cis*-chlordane, 27: *trans*-chlordane, 28: thiamethoxam, 29: *pp'*-DDE, 30: dieldrin, 31: fludioxonil, 32: *op'*-DDT, 33: nitrofen, 34: *pp'*-DDD, 35: *β*-endosulfan, 36: propiconazole, 37: *pp'*-DDT, 38: bifenthrin, 39: fenpropathrin, 40: cyhalothrin, 41: cypermethrin, 42: fenvalerate, and 43: difenoconazole.

Table 1 summarizes the retention times and characteristic mass fragments of the pesticides. The mixture of 43 active ingredients at 0.5 mg L^−1^ was made by each pesticide standard and the total ion chromatogram of the 43 pesticides in standard mixture solution is shown in Figure 1.

### 2.3. Sample Preparation and Measurement Procedure

**Figure 2:**
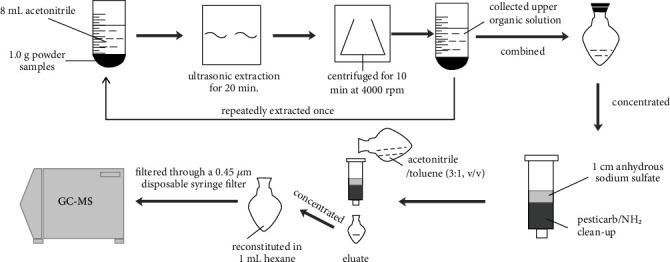
Schematic representation of all steps of the proposed analytical protocol.

The sample preparation procedure is shown in Figure 2. *Schizonepeta tenuifolia* plant samples were ground mechanically to a homogeneous powder and sieved through a no. 50 mesh sieve (355 *μ*m ± 13 *μ*m aperture). 1.0 g powder samples were accurately weighed and placed into a 50 mL polystyrene centrifuge tube, then 8 mL acetonitrile was added. Subsequently, the extraction was centrifuged for 10 min at 4000 rpm with a 3-18K centrifuge (Sigma Co. Germany) after ultrasonic extraction for 20 min. The upper organic solution was collected and the remaining drugs were repeatedly extracted according to the above operation. The mixed organic solution was filtered and concentrated with a vacuum rotary evaporator (40°C).

The Pesticarb/NH_2_ column (500 mg/500 mg/6 mL) was fixed to a support and filled with 1 cm anhydrous sodium sulfate. After being activated with 4 mL acetonitrile/toluene (3 : 1, v/v), the extraction sample was added and eluted with 25 mL acetonitrile/toluene (3 : 1, v/v). The eluant was collected into a heart-shaped flask and concentrated to dryness using a rotary vacuum evaporator (40°C). Finally, the residue was dissolved in 1 mL n-hexane and filtered through a 0.45 *μ*m disposable syringe filter before injecting into an autosampler vial for GC-MS analysis.

## 3. Results and Discussion

### 3.1. Optimization of Extraction Conditions

Due to the complex nature of the matrices in which the target compounds are present and the low detection levels required by regulatory bodies, efficient sample preparation is an important aspect of analytical methods [2]. Extraction and purification are important steps in the sample preparation process. The ultrasonic extractor which is the most commonly used laboratory equipment has good extraction efficiency [17–19]. In this study, the extraction method was optimized using an orthogonal test of L_9_(3^4^), according to different extraction solvent, extraction solvent volume, and the extraction time. A portion (1 mL) of the 1.0 mg L^−1^ standard mixture solutions was added to 1.0000 g of the *Schizonepeta tenuifolia*. For each experimental trial, three replicate experiments were performed. The selected conditions and the results of the orthogonal test are shown in Table 2. Table 2 shows which were found with higher recovery and which is a satisfying recovery under conditions of 16 mL acetonitrile and extraction for 20 min—considered to save time and reduce the solvent. The order of the effect on the recovery was extraction solvent > extraction time > solvent volume. As a result, we applied the best optimized conditions at A_1_B_2_C_2_ (i.e., acetonitrile as extraction solvent, extraction solvent volume of 16 mL, extraction time of 20 min) in the following experiments.

### 3.2. Optimization of Clean-Up Conditions

**Figure 3:**
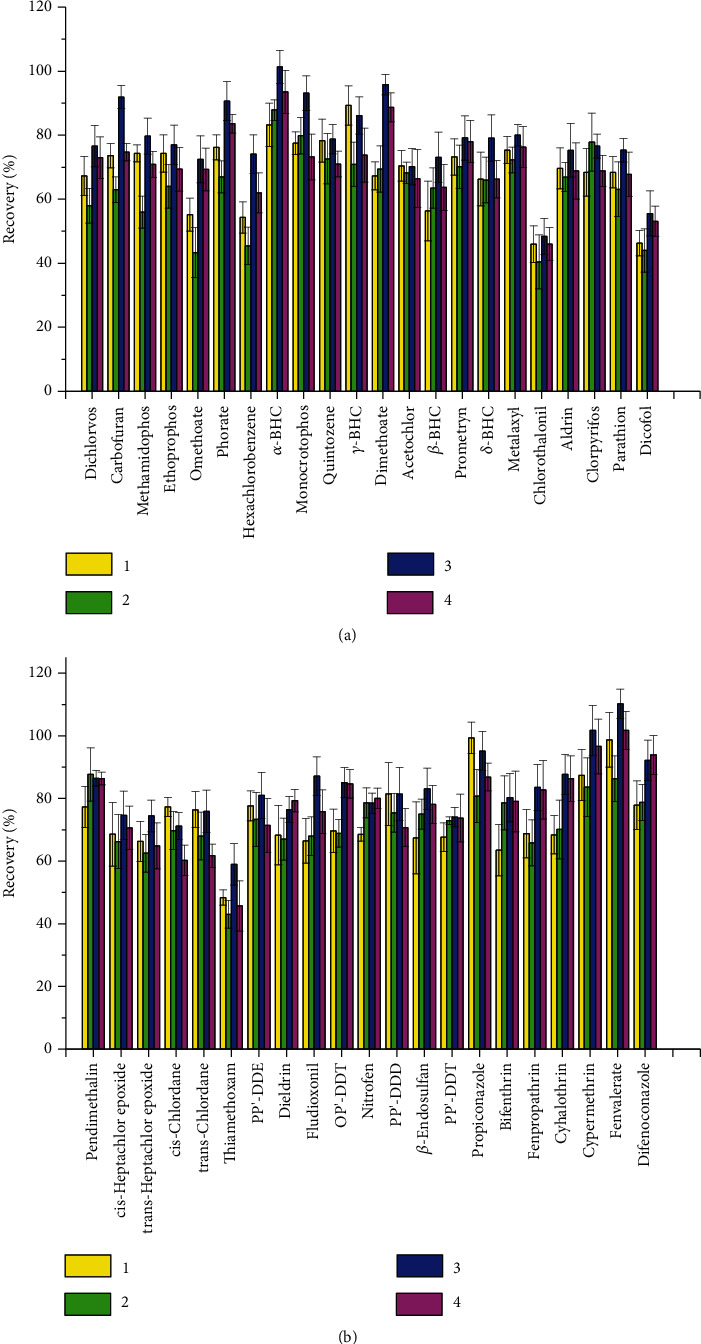
Optimization of the clean-up conditions (a) 1 to 22 pesticides and (b) 23 to 43 pesticides.

During the extraction of *Schizonepeta tenuifolia* samples, not only the analyses are isolated. There are different types of interfering compounds, mainly volatile oil, monoterpenoids and pigments and others [20], which get coextracted. The use of coextraction leads to an unsatisfactory peak shape and an increased or inhibited response, which adversely affects the quantification [21]. Therefore, another analytical step is needed, i.e., extract purification. SPE sorbents, used in dispersive form or packed in a cartridge, are demonstrated suitable to a wide variety of food and agricultural products when appropriate adsorbing/sorbent materials are selected [22–24]. The current study compared three different SPEs, namely, Florisil, TPT, and Pesticarb/NH_2_, on their adsorption capacity for 43 pesticide, the selected conditions are shown in Table 3. The comparison results of various clean-up conditions are shown in Figure 3. Florisil and Pesticarb/NH_2_ do a better job of removing additional matrix components from the extracts than TPT, but Pesticarb/NH_2_ adsorbs more pigments and gets a higher recovery. Therefore, Pesticarb/NH_2_ was chosen as the adsorbent in the extract purification process. Meanwhile, the proportion of acetonitrile/toluene was optimized. The results showed that the recoveries decreased when the proportion of acetonitrile increased. When the samples were eluted with acetonitrile/toluene (3 : 1, v/v), the interference coextractives can be efficiently removed and the recoveries for most pesticides are in the range of 70%–120%.

### 3.3. Validation of the Method

To validate the developed analytical method for 43 pesticides in *Schizonepeta tenuifolia*, linearity of calibration curve, LOD, LOQ, accuracy (recovery %), and precision (relative standard deviation or RSD %) experiments were performed.

#### 3.3.1. Linearity of Calibration Curve, LOD, and LOQ

Linearity was evaluated by matrix-matched calibration curves prepared by spiking 43 pesticides standards (50, 100, 200, 500, 1000 *μ*g L^−1^) in *Schizonepeta tenuifolia.* Based on the result, all the 43 pesticides showed a good linearity with the coefficients of determination (*R*^2^) over than 0.995 (Table 4), which was conducive to accurate quantity. The LOD and LOQ of the method were determined as the minimum detectable concentrations of analyte with signal-to-noise (*S*/*N*) ratio exceeding 3 and 10 by sequential injection of decreasing levels of spiked samples. With this present method, pesticide analyses presented LOD and LOQ in the range of 0.0011–0.0135 mg kg^−1^ and 0.0038–0.0451 mg kg^−1^, respectively. The LOD and LOQ for the test pesticides are presented in Table 5. Compared with the maximum residue limits (MRLs, 0.01–5.0 mg kg^−1^) set by the European Union (EU), United States Environmental Protection Agency (EPA), and Japan [25], the LODs of all pesticides using this method are lower and the determination of pesticide residues would be achieved.

#### 3.3.2. Accuracy and Precision

It is generally known that recovery (%) and relative standard deviations (RSDs) represented the accuracy and precision of the quantitative analysis methods. The recovery experiments were performed to estimate the accuracy of the method with three spiked levels (0.2 mg kg^−1^, 0.5 mg kg^−1,^ and 1 mg kg^−1^) in six replicates. As shown in Table 5, the recovery of most pesticide residues was in the range of 70%–120% with peak area RSDs of 1.32%–13.91%, except Chlorothalonil, Thiamethoxam, and Dicofol. The results demonstrate that the method has a good accuracy and meets the EU's criteria of method validation procedures (SANTE, 2017) [26]. The optimized method is reliable for the simultaneous detection of 43 pesticide residues in *Schizonepeta tenuifolia*. On the other hand, regarding the problem of the low recovery rate of Chlorothalonil, Thiamethoxam, and Dicofol, with no obvious experimental evidences obtained to explain the phenomenon, some instrumental condition variations, analytical error, decomposition of target in blank extract, and any other mechanisms were believed to be linked [27, 28]. The other ways should be investigated to improve the recovery of them in our future work.

#### 3.3.3. Evaluation Method

The comparative study was accomplished between the present technique and some reported methods that the pesticides were detected in *Schizonepeta tenuifolia* or other CHMs. A analytical method was reported by Wu et al. [29] in which just 9 OCPs and PYRs were determined in *Schizonepeta tenuifolia* samples, and the method has the disadvantages of low applicability and the types of pesticide limited. In the present method, the 47 pesticides which most likely to be detected in *Schizonepeta tenuifolia* were selected from the research literature, and they include the four classes mentioned above. Furthermore, the determination of samples is prone to false positive by GC-ECD or GC-FPD.

Besides, Liang et al. [30] developed a method in 2017 in which 33 pesticides in Ginseng samples were detected by gas chromatography mass spectrometry (GC-MS) detector. This method LODs of 33 pesticides ranged from 5 to 25 mg kg^−1^, and RSD is higher than 15%. Compared with the reported method, the LODs of the present method (ranging from 1.1 to 13.5 mg kg^−1^) are comparable to them; however, RSD are lower than 15% which demonstrates that the present method has better sensitivity.

### 3.4. Sample Analysis

Thirty samples were collected from Hebei province, where it is the main producing area of *Schizonepeta tenuifolia* in China. These samples were determined by the established method, and the results are shown in Table 6. From the analytical results, omethoate, monocrotophos, and dimethoate were detected in one sample, while cyhalothrin and parathion were found in 5 and 2 samples, respectively. The contents of detected pesticides in *Schizonepeta tenuifolia* samples were lower than the MRL of European food standard. The pesticide residues in *Schizonepeta tenuifolia* samples may originate from the environmental soil or the administration for controlling pests.

## 4. Conclusions

In this study, a rapid sample preparation for multiresidues detection in *Schizonepeta tenuifolia* was developed by dispersive solid-phase extraction technology. Optimum extraction methods were identified for *Schizonepeta tenuifolia* sample (acetonitrile as extraction solvent, extraction solvent volume of 16 mL, and extraction time of 20 min) and purification (Pesticarb/NH2 as SPE sorbents, eluted with acetonitrile/toluene (3 : 1, v/v) 25 mL) steps. A validation procedure was performed, which showed good results for suitability, recovery, and repeatability. The developed method was applied to the determination of 30 *Schizonepeta tenuifolia* samples, and some pesticides were detected, which demonstrated that it is essential to constantly monitor pesticide residues in *Schizonepeta tenuifolia*. To our knowledge, this approach was first applied to the multiresidue analysis in *Schizonepeta tenuifolia*. The good performance of this method confirmed that it has a strong potential for its application in the monitoring of pesticide residues in *Schizonepeta tenuifolia*.

## Figures and Tables

**Table 1:** Retention times, target ions, and qualifier ions of the selected pesticides.

Pesticide	Retention time	Target ion	Qualifier ion 1	Qualifier ion 2	Qualifier ion 3
Dichlorvos	5.53	109	185	220	
Carbofuran	6.03	164	122	123	
Methamidophos	6.18	94	95	126	
Ethoprophos	10.60	158	200	242	
Omethoate	11.58	156	110	126	
Phorate	11.63	121	231	260	
Hexachlorobenzene	12.07	284	286	282	
*α-*BHC	12.40	219	183	221	254
Monocrotophos	12.96	127	109	192	
Quintozene	13.50	237	249	295	
*γ*-BHC	13.87	183	219	221	254
Dimethoate	14.03	87	93	125	
Acetochlor	15.07	146	162	223	
*β*-BHC	15.12	219	217	181	254
Prometryn	16.14	241	184	226	
*δ*-BHC	16.22	219	181	217	254
Metalaxyl	16.42	206	234	249	
Chlorothalonil	16.52	266	231	194	
Aldrin	16.52	263	265	329	293
Chlorpyrifos	17.31	314	258	286	
Parathion	17.98	291	186	235	
Dicofol	18.60	139	141	252	250
Pendimethalin	18.81	252	220	162	
*cis*-Heptachlor epoxide	19.00	353	351	355	
*trans*-Heptachlor epoxide	19.34	353	351	355	
*cis*-Chlordane	20.12	373	375	377	
*trans*-Chlordane	20.53	373	375	377	
Thiamethoxam	20.88	182	212	247	
*pp'*-DDE	21.73	318	316	246	248
Dieldrin	22.10	263	277	345	380
Fludioxonil	23.39	248	127	154	
*op'*-DDT	23.50	235	237	165	199
Nitrofen	23.60	283	139	202	
*pp'*-DDD	23.89	235	237	165	199
*β*-Endosulfan	24.21	241	265	339	
Propiconazole	24.62	259	173	261	
*pp'*-DDT	24.65	235	237	165	246
Bifenthrin	24.92	181	165	166	
Fenpropathrin	25.75	181	265	349	
Cyhalothrin	26.40	181	197	141	
Cypermethrin	29.88	181	152	180	
Fenvalerate	32.06	167	181	225	419
Difenoconazole	34.88	323	325	265	

**Table 2:** Design and results of orthogonal test.

Trial no.	Factor			Average recovery (%)
	A^a^	B^b^	C^c^	
1	1	1	1	79.6
2	1	2	2	84.6
3	1	3	3	83.2
4	2	1	2	78.4
5	2	2	3	78.3
6	2	3	1	70.6
7	3	1	3	69.4
8	3	2	1	67.3
9	3	3	2	70.5
*K*_*1*_	82.5	75.8	72.5	
*K*_*2*_	75.8	76.8	77.8	
*K*_*3*_	69.1	74.8	77.0	
Range	13.4	2.0	5.3	

*K*_*i*_, mean effect of each factor at level *i* (*i* = 1, 2, 3). ^a^ Factor A, extraction solvent; level 1, acetonitrile; level 2, petroleum ether; level 3, acetone. ^b^ Factor B, volume; level 1, 10 mL; level 2, 16 mL; level 3, 20 mL. ^c^ Factor C, extraction time; level 1, 15 min; level 2, 20 min; level 3, 25 min.

**Table 3:** Experimental program of clean-up.

No.	Type of SPEs	Eluant
1	Florisil	Hexane : ethyl acetate (85 : 15,v/v)
2	TPT	Acetonitrile : methylbenzene (3 : 1,v/v)
3	Pesticarb/NH_2_	Acetonitrile : methylbenzene (3 : 1,v/v)
4	Pesticarb/NH_2_	Acetonitrile : methylbenzene (85 : 15,v/v)

**Table 4:** Regression equations, determination coefficients (*R*^2^), LODs, and LOQs of pesticides.

Pesticide	Regression equation	*R* ^2^	LOD (mg kg^−1^)	LOQ (mg kg^−1^)
Dichlorvos	*Y* = 6.88 × 10^4^*X*－4 313.8	0.996 3	0.001 8	0.005 8
Carbofuran	*Y* = 3.18 × 10^4^*X*－765	0.999 4	0.003 9	0.012 9
Methamidophos	*Y* = 4.90 × 10^3^*X*－664	0.995 9	0.012 2	0.040 7
Ethoprophos	*Y* = 3.79 × 10^4^*X*－1 022.7	0.999 4	0.002 1	0.007 0
Omethoate	*Y* = 1.73 × 10^3^*X*－553.6	0.998 4	0.013 5	0.045 1
Phorate	*Y* = 3.49 × 10^4^*X*－1 189.7	0.997 5	0.002 0	0.006 7
Hexachlorobenzene	*Y* = 3.87 × 10^4^*X*－765.3	0.997 8	0.001 1	0.003 8
*α-*BHC	*Y* = 1.78 × 10^4^*X*－605.8	0.997 1	0.002 4	0.008 0
Monocrotophos	*Y* = 1.35 × 10^4^*X*－2 173.5	0.999 3	0.011 1	0.037 1
Quintozene	*Y* = 1.03 × 10^4^*X*－514.6	0.998 4	0.001 2	0.003 9
*γ*-BHC	*Y* = 2.26 × 10^4^*X*－969.5	0.996 7	0.002 3	0.007 7
Dimethoate	*Y* = 2.93 × 10^4^*X*－2 078.2	0.995 3	0.003 4	0.011 3
Acetochlor	*Y* = 3.75 × 10^4^*X*－135 5	0.998 6	0.003 9	0.012 9
*β*-BHC	*Y* = 1.04 × 10^4^*X*－386.7	0.997 9	0.001 6	0.005 5
Prometryn	*Y* = 4.61 × 10^4^*X*－1 485.4	0.998 2	0.004 8	0.015 8
*δ*-BHC	*Y* = 1.68 × 10^4^*X*－596.9	0.998 2	0.001 7	0.005 7
Metalaxyl	*Y* = 2.65 × 10^4^*X*－1 004.2	0.998 8	0.002 6	0.008 7
Chlorothalonil	*Y* = 6.88 × 10^4^*X*－11 033.2	0.996 4	0.038 0	0.012 7
Aldrin	*Y* = 1.83 × 10^4^*X*－666.9	0.999 4	0.003 8	0.012 5
Chlorpyrifos	*Y* = 1.94 × 10^4^*X*－976.2	0.995 2	0.002 2	0.007 3
Parathion	*Y* = 1.47 × 10^4^*X*－859.9	0.995 6	0.002 8	0.009 2
Dicofol	*Y* = 1.00 × 10^6^*X*－2 599.8	0.997 6	0.003 0	0.009 9
Pendimethalin	*Y* = 3.45 × 10^4^*X*－2 108.9	0.997 1	0.007 3	0.024 4
*cis*-Heptachlor epoxide	*Y* = 1.94 × 10^4^*X*－690.5	0.997 5	0.001 9	0.006 3
*trans*-Heptachlor epoxide	*Y* = 2.86 × 10^3^*X*－64.6	0.997 6	0.001 2	0.004 1
*cis*-Chlordane	*Y* = 3.27 × 10^4^*X*－1 264.7	0.995 6	0.001 9	0.006 2
*trans*-Chlordane	*Y* = 2.49 × 10^4^*X*－807.7	0.997 5	0.001 2	0.004 0
Thiamethoxam	*Y* = 1.00 × 10^4^*X*－461	0.998 6	0.002 7	0.009 2
*pp'*-DDE	*Y* = 4.88 × 10^4^*X*－1 159.8	0.996 9	0.006 2	0.020 7
Dieldrin	*Y* = 8.88 × 10^3^*X*－347.5	0.997 1	0.003 0	0.010 1
Fludioxonil	*Y* = 2.25 × 10^4^*X*－936.1	0.998 4	0.010 0	0.033 3
*op'*-DDT	*Y* = 6.47 × 10^4^*X*－3 256.6	0.999 4	0.005 1	0.016 9
Nitrofen	*Y* = 1.95 × 10^4^*X*－1 081.4	0.995 3	0.001 4	0.004 7
*pp'*-DDD	*Y* = 9.49 × 10^4^*X*－2 277.2	0.997 9	0.003 6	0.011 9
*β*-Endosulfan	*Y* = 3.04 × 10^3^*X*－17.7	0.998 5	0.001 3	0.004 4
Propiconazole	*Y* = 2.19 × 10^4^*X*－882.9	0.998 3	0.001 1	0.037 0
*pp'*-DDT	*Y* = 4.84 × 10^4^*X*－1 800.2	0.999 0	0.002 6	0.008 5
Bifenthrin	*Y* = 1.69 × 10^4^*X*－2 085.6	0.998 1	0.007 8	0.026 0
Fenpropathrin	*Y* = 3.28 × 10^4^*X*－1 073.1	0.996 4	0.005 8	0.019 3
Cyhalothrin	*Y* = 4.81 × 10^4^*X*－2 338.9	0.995 8	0.001 5	0.004 9
Cypermethrin	*Y* = 1.78 × 10^4^*X*－963.2	0.997 7	0.001 9	0.006 3
Fenvalerate	*Y* = 2.24 × 10^4^*X*－1 585.6	0.995 3	0.001 3	0.004 3
Difenoconazole	*Y* = 5.74 × 10^4^*X*－2 995.7	0.997 6	0.005 3	0.017 7

**Table 5:** Results of recovery test (*n* = 6).

Pesticide	0.2 mg kg^−1^		0.5 mg kg^−1^		1 mg kg^−1^	
	Recovery (%)	RSD (%)	Recovery (%)	RSD (%)	Recovery (%)	RSD (%)
Dichlorvos	80.97	7.50	75.92	8.61	88.10	7.36
Carbofuran	90.64	4.20	89.77	2.96	96.88	1.58
Methamidophos	76.33	3.48	70.33	5.79	72.94	7.75
Ethoprophos	72.60	8.01	75.68	9.03	70.42	8.77
Omethoate	75.32	6.86	84.91	7.90	86.87	8.45
Phorate	92.80	4.17	87.69	3.33	85.60	7.15
Hexachlorobenzene	77.82	6.30	76.81	8.17	71.93	8.33
*α-*BHC	117.29	8.33	105.85	6.39	109.63	8.35
Monocrotophos	92.94	3.78	97.58	7.31	92.91	5.78
Quintozene	72.21	9.29	79.66	5.04	75.42	6.11
*γ*-BHC	79.29	7.74	81.26	10.38	70.11	8.32
Dimethoate	100.02	4.39	82.25	5.51	84.30	3.79
Acetochlor	70.43	6.79	70.88	12.86	72.94	7.76
*β*-BHC	89.08	11.60	77.79	9.23	73.87	10.61
Prometryn	70.43	8.07	80.51	8.20	83.98	8.22
*δ*-BHC	79.12	10.61	75.13	7.76	74.02	9.75
Metalaxyl	71.11	5.99	84.38	7.59	74.13	4.53
Chlorothalonil	48.43	11.77	49.33	10.43	54.93	10.32
Aldrin	71.75	8.96	82.31	10.68	79.65	10.48
Chlorpyrifos	83.33	8.92	74.89	6.45	71.43	2.45
Parathion	79.68	2.36	77.32	8.93	75.94	4.86
Dicofol	45.61	8.75	57.42	8.29	61.89	11.38
Pendimethalin	81.18	8.07	73.90	2.76	76.90	3.30
*cis*-Heptachlor epoxide	78.91	12.87	73.92	9.41	75.92	10.17
*trans*-Heptachlor epoxide	78.42	8.11	76.51	9.56	75.81	6.63
*cis*-Chlordane	75.11	4.01	85.17	2.14	79.27	11.71
*trans*-Chlordane	74.01	7.76	70.45	5.25	80.58	8.41
Thiamethoxam	57.89	4.23	58.91	13.61	65.53	10.06
*pp'*-DDE	70.37	6.80	81.90	10.55	74.21	9.95
Dieldrin	75.80	12.55	74.28	4.81	72.88	5.70
Fludioxonil	83.25	8.56	78.20	8.89	77.78	7.94
*op'*-DDT	80.61	8.63	72.20	6.28	85.19	5.60
Nitrofen	92.22	2.36	92.81	1.38	91.32	1.32
*pp'*-DDD	80.77	12.46	72.65	8.28	83.78	10.14
*β*-Endosulfan	83.12	13.86	81.89	7.39	87.13	9.91
Propiconazole	94.19	4.84	87.21	8.73	95.53	3.25
*pp'*-DDT	73.68	6.82	74.75	5.90	75.45	8.22
Bifenthrin	70.34	11.64	83.63	11.55	74.27	10.37
Fenpropathrin	84.31	9.15	80.61	12.81	82.45	8.93
Cyhalothrin	86.32	7.07	88.28	8.23	99.06	9.48
Cypermethrin	99.53	10.22	91.62	13.91	87.47	9.06
Fenvalerate	108.70	9.83	101.28	12.93	103.91	8.35
Difenoconazole	103.65	12.29	112.35	9.94	104.65	7.10

**Table 6:** Pesticide residues (mg kg^−1^) determined in Schizonepeta tenuifolia samples.

Pesticide	No. of positive samples	Min.	Max.	MRL
Cyhalothrin	5	0.056	0.680	1
Omethoate	1	ND^a^	0.065	0.1
Monocrotophos	1	ND	0.068	0.1
Dimethoate	1	ND	0.099	0.1
Parathion	2	0.081	0.088	0.5

^a^ Not detectable or lower than limit of detection.

## Data Availability

The data used to support the findings of this study are included within the article.
